# Cardiac involvement in cardiac AL amyloidosis as measured by equilibrium contrast cardiovascular magnetic resonance

**DOI:** 10.1186/1532-429X-14-S1-P174

**Published:** 2012-02-01

**Authors:** Sanjay M Banypersad, Daniel Sado, Andrew Flett, Simon D Gibbs, Jennifer H Pinney, Viviana Maestrini, Steven K White, Jason Dungu, Philip N Hawkins, James Moon

**Affiliations:** 1 CMR, Heart Hospital, London, UK; 2 National Amyloidosis Centre, Royal Free Hospital, London, UK

## Background

Involvement of the heart drives prognosis in Systemic AL Amyloidosis, predicting outcome and influencing therapeutic options. Current methods of cardiac assessment do not allow formal quantification of the amyloid load. We used Equilibrium Contrast Cardiovascular Magnetic Resonance (EQ-CMR) to measure the interstitial compartment of the heart by measuring the myocardial contrast volume of distribution, VDm.

## Methods

Patients with systemic AL amyloidosis undergoing routine work up at the National Amyloidosis Centre were recruited (n=54, 35 males, 19 females, mean age 64) and underwent EQ-CMR and conventional CMR including late enhancement to measure VDm and standard cardiac work-up including ECG, echocardiography, biomarkers (BNP, troponin) and functional assessment (6 minute walk test, 6MWT, where permitted by autonomic neuropathy). Results were compared to normal controls. Conventional assessment ranked cardiac involvement as definite, probable and none.

## Results

VDm was significantly higher in patients than normal controls (0.25 vs 0.40, P < 0.001). This tracked conventional cardiac assessment (none, probable, definite corresponded with a VDm of 0.276 vs 0.342 vs 0.488, P<0.005), respectively. VDm correlated with cardiac parameters by echo (e.g. TDI S-wave R^2^ 0.24, P=0.001) and conventional CMR (e.g. indexed LV mass R^2^ 0.31, P<0.001). Significant correlations were also seen with BNP (R^2^ 0.2, P<0.005) and Trop T (R^2^ 0.26, P<0.01). VDm was associated with ECG abnormalities and tracked small QRS voltages (R^2^ 0.43, P<0.001). A higher VDm trended towards a lower 6-minute walk test outcome (R^2^ 0.1, P=0.09)

## Conclusions

The measurement of the myocardial interstitial compartment (VDm) using EQ-CMR in systemic AL amyloidosis has the potential to be the first quantitative test for cardiac amyloid deposition.

## Funding

GSK

**Figure 1:**
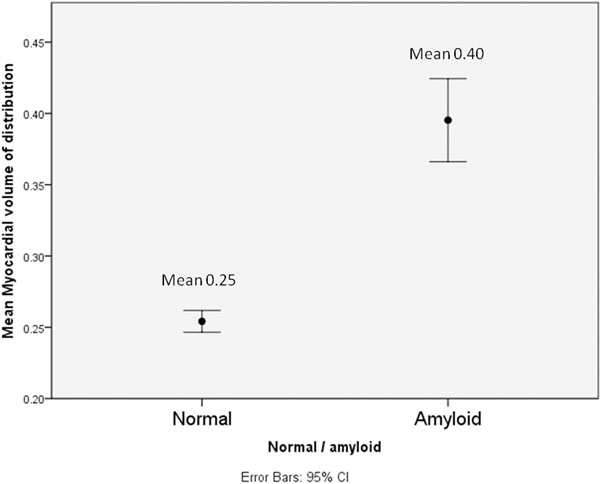
showing the mean myocardial volume of distributions (and 95% confidence intervals) for normals and patients with systemic AL amyloidosis (P<0.001).

**Figure 2:**
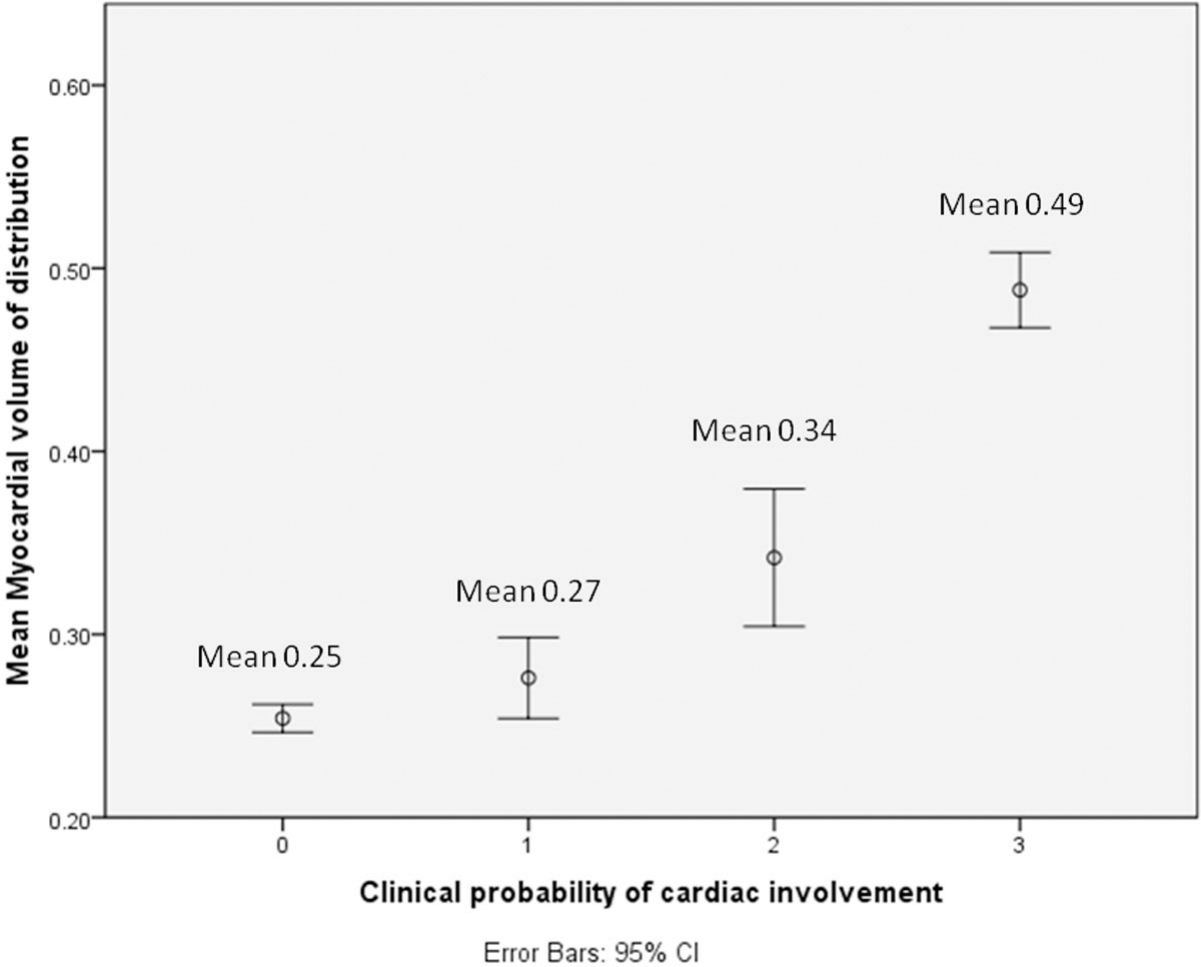
compares the mean VDm between normals (group 1) and Systemic AL Amyloidosis patients with : No suspected cardiac involvement (group 2, P=0.44), possible cardiac involvement (group 3, P<0.001) and definite cardiac involvement (group 4, P<0.001).

